# Prognostic importance of circulating epidermal growth factor-like domain 7 in patients with metastatic colorectal cancer treated with chemotherapy and bevacizumab

**DOI:** 10.1038/s41598-017-02538-x

**Published:** 2017-05-24

**Authors:** Torben Frøstrup Hansen, Rikke Fredslund Andersen, Dorte Aalund Olsen, Flemming Brandt Sørensen, Anders Jakobsen

**Affiliations:** Danish Colorectal Cancer Center South, Vejle Hospital, Institute of Regional Health Research, University of Southern Denmark, Odense, Denmark

## Abstract

High tumor expression of epidermal growth factor-like domain 7 (EGFL7) has been associated with a poor prognosis in colorectal cancer. The aim of the current study was to investigate the possible prognostic impact of circulating EGFL7 (cir-EGFL7), combined with single nucleotide polymorphism (SNP) analyses, in patients with metastatic colorectal cancer (mCRC) treated with first line chemotherapy and bevacizumab. A total of 88 patients were included. Serum was collected prior to treatment initiation, at first evaluation after 3 weeks, and at progression. Cir-EGFL7 was analysed by the enzyme-linked immunosorbent assay (ELISA) technique. The SNPs were analysed by real-time qPCR based on DNA from whole blood. Endpoints were response rate (RR), progression free survival (PFS), and overall survival (OS). Cir-EGFL7 decreases after administration of chemotherapy plus bevacizumab. Baseline levels of cir-EGFL7 were significantly related to PFS and OS, p = 0.0431 and p = 0.0017, respectively, with increasing cir-EGFL7 levels associated with a worse prognosis. Circulating EGFL7 was not associated with RR. The SNP analyses revealed a significant relationship between rs1051851 and OS, p = 0.030. This study demonstrates that cir-EGFL7 changes during treatment with chemotherapy plus bevacizumab and that baseline levels and genetic variations may influence the overall prognosis of patients with mCRC. The findings call for further validation.

## Introduction

Patients diagnosed with metastatic colorectal cancer (mCRC) have a poor prognosis. Recent years, however, have witnessed an increase in median overall survival (OS) beyond two years as recently demonstrated in the TRIBE study^1^. This may, among other reasons, be attributable to individualized treatment strategies aiming at optimal benefit of all available treatment modalities in every line of treatment. Despite the recent increase in OS, the benefit from first line treatment alone has not changed over the same period calling for increased focus on upfront treatment strategies.

Targeting angiogenesis has been a standard treatment modality in mCRC over a decade and new anti-angiogenic drugs are approved every year. While the understanding of the molecular sub-classification of colorectal cancer (CRC) is improving, the field of anti-angiogenic treatments is still characterized by the lack of validated biomarkers.

In contrast to normal blood vessels, the endothelial cells (ECs) of tumor associated blood vessels are highly active and contribute to the shedding of cell components into the circulation, including pro-angiogenic factors^2^. This could reflect tumor load in general and may change during the course of treatment. We have previously shown that changes in the levels of EC specific microRNA-126 (miRNA-126) during treatment may be predictive as to benefit of first line chemotherapy and bevacizumab in patients with mCRC^3^. MicroRNA-126 is transcribed from the epidermal growth factor-like domain 7 (*EGFL7*) gene^4^, and the EGFL7 protein is secreted by activated ECs, a process almost non-existing in normal quiescent blood vessels^5–8^. In the extracellular matrix, EGFL7 supports EC adhesion and migration^7, 9^, protects the ECs from apoptosis^10^, and is important for tube formation^7^ and guiding of endothelial sprouts^9^. Recent studies have suggested a relationship between efficacy of first line treatment and tumor expression of EGFL7^11, 12^, but the role of circulating EGFL7 (cir-EGFL7) in this context has so far not been explored. The possible influence of variations in the *EGFL7* gene also calls for elucidation.

The aim of the current study was to investigate the possible prognostic impact of cir-EGFL7, combined with single nucleotide polymorphism (SNP) analyses, in patients with mCRC treated with first line chemotherapy and bevacizumab.

## Results

### Patient characteristics

The main patient characteristics of the included 88 patients are summarised in Table 1. At the time of cir-EGFL7 analysis, *i.e*. at a median follow-up of 28.9 months, the median progression free survival (PFS) was 7.6 months (95% confidence interval (CI): 7.0–9.2 months), whereas the median OS was 20.9 months (95% CI: 18.4–23.8).

**Table 1:** Patient characteristics.

	N^a^ = 88
Gender	
Male	53 (60)
Female	35 (40)
Age (years)^*^	
Mean (SD^b^)	65 (10)
Range	32–79
>Mean	54 (61)
≤Mean	34 (39)
ECOG PS^c^	
0	57 (65)
1–2	31 (35)
Tumor resection^**^	
Yes	28 (32)
No	60 (68)
Localization	
Colon	53 (60)
Rectum	33 (38)
Synchronous	2 (2)
Metastatic sites	
1	39 (44)
≥2	49 (56)
Adjuvant chemotherapy^***^	
Yes	4 (5)
No	82 (95)
*RAS/RAF* ^***,****^	
Wild type	35 (40)
Mutated	49 (56)
Unknown	4 (5)
Further lines of treatment	
2. line	59 (67)
3. line	28 (32)
≥4. line	11 (13)

N^a^, Number; SD^b^, Standard deviation; ECOG PS^c^: Eastern Cooperative Oncology Group performance status.

Not all sums of percentages equal 100% due to rounding of data.

^*^Age at start of treatment.

^**^Primary tumor previously resected.

^***^Data registration incomplete.

^****^Includes KRAS, NRAS, and BRAF mutational status.

### Circulating epidermal growth factor-like domain 7

**Figure 1:**
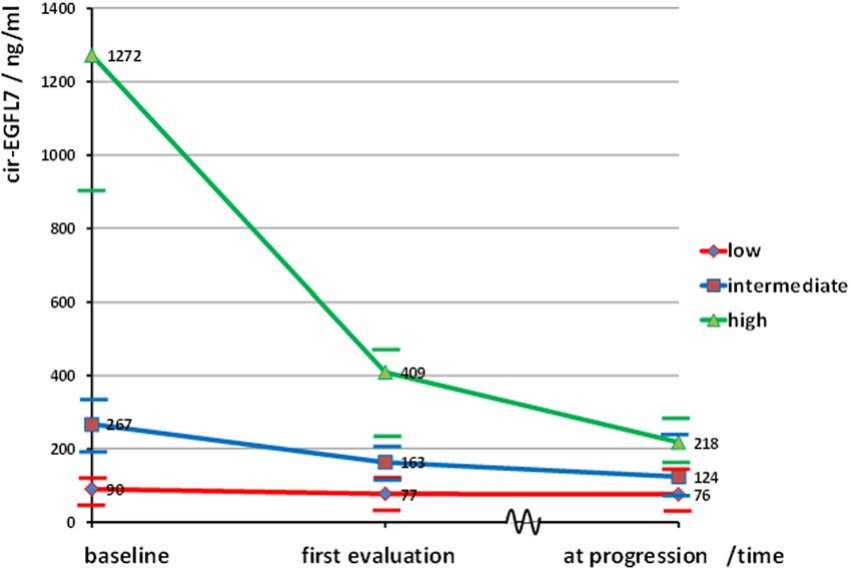
Median circulating epidermal growth factor-like domain 7 (cir-EGFL7) according to baseline levels (lowest third, intermediate third, and highest third) at baseline, first evaluation, and progression. Horizontal lines mark the respective upper and lower limits of the 95% confidence intervals (CI). The upper limit (2003 ng/ml) of the 95% CI for the high cir-EGFL7 levels at baseline is censored for graphical reasons, but not from the analyses. The broken time line between first evaluation and progression indicates that this time period varies between the patients. The differences between the medians for the low, intermediate, and high groups were significant at baseline and at first evaluation but not at time of progression (p < 0.05).

Treatment with chemotherapy plus bevacizumab resulted in a significant reduction in the cir-EGFL7 levels. The median concentration at baseline was 269 ng/ml (95% CI: 170–396), at the first assessment after three weeks it was 171 ng/ml (95% CI: 118–212), and later on changes were rather modest up to the progression level of 130 ng/ml (95% CI: 101–182), p = 0.003. Dividing the patients into three groups (tertiles) according to baseline levels revealed that decreasing cir-EGFL7 during treatment was primarily a characteristic of the patients with high baseline levels (Fig. 1). Baseline cir-EGFL7 was significantly lower in patients who had undergone primary tumor resection 136 ng/ml (95% CI: 86–227) compared to those with the primary tumor *in situ* 504 ng/ml (95% CI: 203–872), p = 0.0001. The same applies to patients with a rectal cancer compared to colon cancer (p = 0.0108), and the 15 patients that underwent R0 resection after treatment initiation compared to those who did not (p = 0.0114, Supplementary Table 1). Cir-EGFL7 was not related to RAS/RAF status (p = 0.56, Supplementary Table 1). Addressing liver limited disease only, the potential candidates for R0 resection showed a similar association with a median EGFL7 of 117 ng/ml (95% CI: 68–227) in patients who underwent resection later compared to 400 ng/ml (95% CI: 159–872) in those remaining unresectable, p = 0.014.

**Figure 2:**
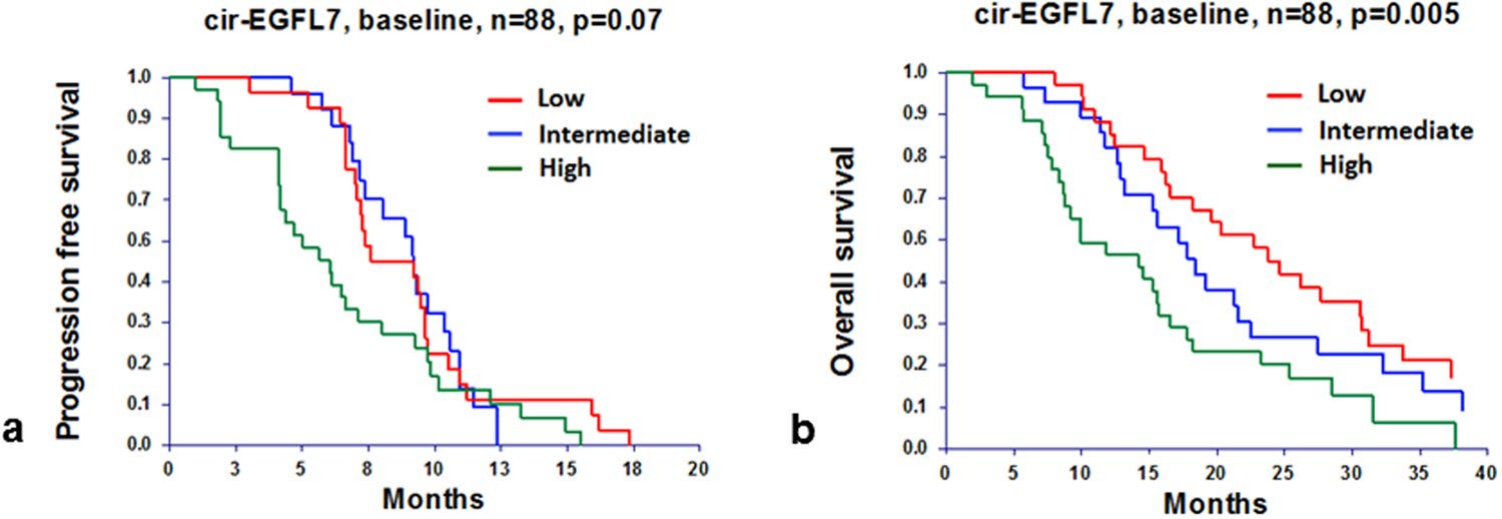
Progression free (**a**) and overall (**b**) survival according to circulating epidermal growth factor-like domain 7 (cir-EGFL7) levels at baseline.

Neither the baseline level of cir-EGFL7 nor its dynamics during treatment was related to treatment response (Supplementary Table 2). When grouped according to level, baseline cir-EGFL7 demonstrated a non-significant relationship with PFS in the log rank test as illustrated in Fig. 2a, whereas the Cox regression analysis indicated a significantly worse PFS of patients with a high baseline level (Table 2).

**Table 2:** Cox regression analysis, progression free survival (n = 84 in the multiple analysis).

	simple analysis			multiple analysis		
	HR^a^	95% CI^b^	p-value	HR	95% CI	p-value
Gender						
Female	0		0.7444			
Male	1.0825	0.6721–1.7437				
Age (years)^*^	1.0104	0.9849–1.0366	0.4275			
ECOG PS^c^						
0	1		0.0608	1		0.1351
1–2	1.5822	0.9794–2.5561		1.4661	0.8876–2.4216	
Tumor resection						
No	1		0.3908			
Yes	0.7906	0.4623–1.3520				
Localization^**^						
Rectum	1		0.2160			
Colon	1.3619	0.8349–2.2214				
Metastatic sites						
1	1		0.3631			
≥2	1.2528	0.7708–2.0360				
Adjuvant chemotherapy						
No	1		0.8141			
Yes	0.8851	0.3198–2.4494				
*RAS/RAF* ^d***^						
Wild type	1		**0.0035**	1		
Mutated	2.1004	1.2755–3.4588		2.1748	1.3059–3.6218	**0.0028**
cir-EGFL7^e^						
Low	1			1		
Intermediate	1.0877	0.5858–2.0195	0.7901	1.1991	0.6322–2.2741	0.5782
High	1.7955	1.0208–3.1583	**0.0422**	1.8264	1.0165–3.2819	**0.0440**

HR^a^, hazard ratio; CI^b^, confidence interval; ECOG PS^c^, Eastern Cooperative Oncology Group Performance Status; *RAS/RAF* ^d^, KRAS, NRAS, and BRAF mutational status; cir-EGFL7^e^, circulating epidermal growth factor-like domain 7.

^*^Age is included in the analyses as a continuous parameter.

^**^The two patients with synchronous tumors were not included in the analyses.

^***^The four patients with unknown *RAS/RAF* mutational status were not included in the Cox regression analysis leading to N = 84 in the multiple analyses.

Impaired OS was demonstrated of patients with high baseline cir-EGFL7 (Fig. 2b); an association that remained significant after a multiple Cox regression analysis, hazard ratio 2.0331 (95% CI: 1.0077–4.1019), p = 0.0476 (Table 3).

**Table 3:** Cox regression analysis, overall survival (n = 56 in the multiple analysis).

	simple analysis			multiple analysis		
	HR^a^	95% CI^b^	p-value	HR	95% CI	p-value
Gender						
Female	0		0.6134			
Male	0.8858	0.5535–1.4177				
Age (years)^*^	1.0115	0.9954–1.0254	0.1102			
ECOG PS^c^						
0	1		**0.0118**	1		0.3508
1–2	1.8613	1.1479–3.0182		1.3581	0.7140–2.5829	
Tumor resection						
No	1		**0.0335**			
Yes	0.5863	0.3525–0.9750				
Localization^**^						
Rectum	1		0.1319			
Colon	1.5179	0.8820–2.6121				
Metastatic sites						
1	1		**0.0012**	1		0.4672
≥2	2.2790	1.3842–3.7523		1.2628	0.6732–2.3686	
Adjuvant chemotherapy						
No	1		0.8046			
Yes	0.8800	0.3196–2.4228				
*RAS/RAF* ^d,^ ***						
Wild type	1		**0.0003**	1		
Mutated	2.5973	1.5575–4.3313		2.7705	1.4657–5.2368	0.0017
cir-EGFL7^e^						
Low	1			1		
Intermediate	1.4416	0.8025–2.5897	0.2211	1.3950	0.5862–3.3198	0.4518
High	2.4647	1.4038–4.3271	**0.0017**	2.0331	1.0077–4.1019	**0.0476**

HR^a^, hazard ratio; CI^b^, confidence interval; ECOG PS^c^, Eastern Cooperative Oncology Group Performance Status; *RAS/RAF* ^d^, KRAS, NRAS, and BRAF mutational status; cir-EGFL7^e^, circulating epidermal growth factor-like domain 7.

^*^Age is included in the analysis as a continuous parameter.

^**^The two patients with synchronous tumours were not included in the analysis.

^***^The four patients with unknown *RAS/RAF* mutational status were not included in the Cox regression analysis.

The multiple analyses are restricted to patients without prior resection of the primary tumor, due to interaction between tumor resection and cir-EGFL7, leading to N = 54 in the multiple analyses.

### Epidermal growth factor-like domain 7 single nucleotide polymorphisms

The distribution of genotypes in the five analyzed SNPs followed the Hardy-Weinberg equilibrium (Supplementary Table 3). There were no significant relationships between genotype distributions and cir-EGFL7 (Supplementary Table 4).

The response rate of patients harboring the CT genotype of the rs4880118 SNP was significantly higher than the patients with the CC genotype, 10/13 = 77% and 31/70 = 44%, p = 0.038. This relationship, however, did not translate into a significant difference in PFS (Table 4).

**Table 4:** Genotype distribution in relation to treatment response, progression free survival, and overall survival.

SNP^d^	Response (yes/no)		PFS^a^ (medians, 95% CI^b^)/months		OS^c^ (medians, 95% CI)/months	
	(N^e^ = 83^*^)	p-value	(N = 86)	p-value^**^	(N = 86)	p-value^**^
rs^f^7041558						
AA	6/6		8.0 (6.6–8.9)		15.2 (10.0–19.2)	
AG	20/21	0.994	8.8 (7.0–9.3)	0.585	19.6 (15.7–23.2)	0.200
GG	15/15		7.2 (6.6–9.2)		16.2 (12.6–22.7)	
rs9411215						
AA	3/4		7.3 (7.0–11.5)		19.2 (15.2–26.2)	
AG	16/17	0.912	8.8 (6.9–9.4)	0.943	16.5 (11.8–21.6)	0.706
GG	22/21		7.6 (6.2–9.3)		17.1 (14.2–23.2)	
rs4880118						
CC	31/39		7.3 (6.8–9.1)		16.2 (14.6–19.2)	
CT	10/3	**0.038**	9.7 (6.9–9.8)	0.848	24.6 (15.3–27.5)	0.242
TT	0/0					
rs1051851						
GG	26/28		8.8 (6.6–0.3)		21.3 (15.6–27.5)	
GA	15/13	0.547	7.3 (6.8–91)	0.164	15.2 (11.7–17.1)	**0.030**
AA	0/1		13.2		37.6	
rs4880119^b^						
GG	1/2		7.3 (7.3–11.5)		19.2 (15.2–19.2)	
GA	10/14	0.529	7.0 (6.6–9.2)	0.565	14.6 (11.0–18.2)	0.075
AA	30/26		8.0 (6.9–9.3)		19.6 (15.6–23.7)	

PFS^a^, Progression free survival; CI^b^, Confidence interval; OS^c^, Overall survival; SNPd, Single nucleotide polymorphism; N^e^, Number; rs^f^: reference sequence.

^*^Two patients had insufficient DNA for SNP analyses, three patients were not evaluable according to RECIST.

^**^p-values according to the Log-rank test baseline.

The genotype distribution in the rs1051851 SNP demonstrated a significant relationship with OS (p = 0.03) (Table 4).

## Discussion

Recent studies have indicated that high tumor expression levels of EGFL7 may be associated with poor prognosis in several different malignancies^12–16^. Furthermore, we have previously shown a relationship between high expression levels of EGFL7 in CRC tissue and reduced response rate (RR) in the metastatic setting^11, 12^, and antibodies targeting EGFL7 have demonstrated efficacy in early phases of clinical testing^17^. In order to further characterize the clinical value of EGFL7 it is necessary to address it in the circulation and investigate how this pro-angiogenic protein is influenced by disease and treatment.

The present study demonstrated a relationship between high levels of cir-EGFL7 at baseline and poor PFS and OS for patients with mCRC treated with first line chemotherapy and bevacizumab. To the best of our knowledge the study by Fan *et al*. is the only previous study in which EGFL7 has been analysed in blood samples from patients with CRC^13^. They demonstrated an elevated median EGFL7 concentration in serum from patients with different cancers, among these 64 patients with CRC, compared to a reference cohort of healthy donors. The possible association with clinical outcome was not addressed in their study. A publication from 2015 by Liu *et al*. demonstrated significantly higher serum levels of EGFL7 in patients with hepatocellular carcinomas compared to healthy donors^18^. Both studies thus demonstrated elevated cir-EGFL7 in the presence of cancer. The results of the present study are in line with previous tissue-based studies, emphasizing the prognostic disadvantage of high EGFL7 levels, while simultaneously demonstrating this relationship to be accessible through the analysis of blood samples. This approach holds important advantages compared to tissue analysis, including easier sampling and the avoidance of tumor heterogeneity. In regard to the survival analyses, one may argue that the censoring of PFS data from 15 out of 88 patients is problematic. However, since the censored patients are expected to be at lower risk of progression (limited disease and resected) than the remaining patients in the analyses, and given the fact that the majority of the censored patients are from the low (N = 8) and middle (N = 5) EGFL7 groups (baseline), the true difference in PFS based on EGFL7 base-line levels is likely to be bigger^19^ than the one illustrated in the present analyses.

Given this association with PFS and OS, and the similar findings in other malignancies, the lack of relationship with treatment efficacy seems rather surprising. This is especially the case considering our previous studies demonstrating a relationship between RR and EGFL7 tumor tissue expression levels^11, 12^. In addition to multiple methodological differences (formalin fixed paraffin embedded tissue *versus* serum, immunohistochemistry *versus* enzyme-linked immunosorbent assay (ELISA), subjective/semi-quantitative *versus* quantitative assessment, etc.) the angiogenic activity assessed by EGFL7 *in situ* may simply provide a better estimate of tumor responsiveness. Such a composite tumor estimate provides both quantitative information about the blood vessel area and functional input as to the multiple angiogenic processes modulated by EGFL7, e.g. blood vessel maturity that may be linked to chemotherapy uptake^20^. In contrast, cir-EGFL7 is more likely to reflect the overall state of activated tumor angiogenesis from the entire tumor burden in the patient. Upon treatment initiation, which especially targets immature blood vessels, this burden, and its ability to secrete EGFL7, decreases without necessarily providing specific information as to the delivery/vulnerability of the individual tumors in the patient. Upon treatment resistance, these patients may experience tumor regrowth to a larger extent (multiple sites), which may explain the association with OS (intrinsic treatment resistance).

A number of clinical observations from the present study deserve to be discussed in further detail.

First of all it was interesting, that baseline cir-EGFL7 levels of potentially resectable patients were guiding as to later resectability. Patients who underwent resection later during their treatment course presented with significantly lower baseline values compared to the patients that was not resected. In support of this observation are the results presented by Shen *et al*. in a study, where EGFL7 was analyzed in serum from 112 patients with pancreatic cancer^21^. In this study EGFL7 was lower in resectable patients compared to the non-resectable and in patients who underwent resection EGFL7 dropped significantly postoperatively. These observations, of course, needs to be investigated in suitable settings, but the similarities between the results supports their plausibility and the idea of a molecular biomarker instrumental in the upfront selection of patients for intensive treatment is of high clinical relevance.

A second observation concerns tumor load in general. It appears that the initial tumor burden seems to influence the steady state levels of cir-EGFL7, i.e. primary tumor resected or not. Patients with the primary tumor *in situ* presented with significantly higher levels of cir-EGFL7 (approximately 4 fold) than those who had previously undergone tumor resection. This corresponds rather well with basic angiogenic knowledge stating that tumor associated ECs are highly activated, leaky, and release high levels of pro-angiogenic proteins such as EGFL7^2^ as compared to the remaining vascular network, where EGFL7 expression is rather limited in the quiescent ECs^5^.

The third point to be highlighted is in regard to the dynamics of cir-EGFL7. Overall, cir-EGFL7 dropped after initiation of therapy but was maintained at low levels during treatment until progression, suggesting that the acquired treatment resistance to some extent may be EGFL7 independent. This scenario is similar to what has previously been shown for vascular endothelial growth factor A (VEGF-A) during bevacizumab treatment^22^. This specific observation may be highly relevant considering the future possibility of adding anti-EGFL7 antibodies to the treatment regimens, as such a step needs to be guided by the fluctuation of relevant biomarkers, such as cir-EGFL7.

Turning to the genomics, the *EGFL7* associated SNPs analyzed in the present study did not demonstrate any evidence of functionality in regard to cir-EGFL7 levels. The rs4880118 SNP demonstrated a significant relationship with RR, but this difference was not translated to a difference in PFS and hence, the clinical importance of this observation is uncertain. A significant difference in OS was observed when addressing the rs1051851 SNP. However, based on the genotypes it was not clear whether the A- or G-allele determined the survival advantage. These results call for further assessment and validation in larger cohorts in order to determine the potential clinical impact. In 2013, Li *et al*. presented an abstract at the ASCO Annual Meeting (J Clin Oncol 31, 2013 (suppl; abstr 3565)) arguing for a relationship between the *EGFL7* rs1051851 SNP and RR from a pooled cohort of 455 patients with mCRC treated with first line FOLFIRI and bevacizumab, but the original article containing these observations does not seem to have been published, yet. The lack of obvious relationships between the investigated SNPs and treatment outcome was the main reason for not exploring possible associations with underlying haplotypes in the *EGFL7* gene.

In reference to previously published data on this cohort^12^ we found no relationship between tumor expression of EGFL7 and the present cir-EGFL7 data. These comparisons across data furthermore indicate that tumors from patients with the rs9411215 GG genotype presented with a lower EGFL7 tumor expression but this is based on a rather small subsample (N = 40).

In conclusion, this exploratory investigation demonstrates that cir-EGFL7 decreases after the first administration of chemotherapy plus bevacizumab, and how that the baseline level of this angiogenic parameter is of prognostic importance in patients with mCRC. These findings along with the possible influence of the rs1051851 SNP on OS call for further validation.

## Methods

Reporting in this study is in accordance with the REMARK^23^ and BRISQUE^24^ criteria. The analysing of angiogenesis related SNPs and proteins was pre-specified in the study protocol.

### Study population

This study prospectively included 88 patients between March 2010 and October 2013 with histologically verified mCRC deemed unresectable at baseline, measurable disease according to the Response Evaluation Criteria In Solid Tumours (RECIST 1.1)^25^, and planned for first line chemotherapy combined with bevacizumab. Patients having completed adjuvant chemotherapy more than 6 months before enrolment were accepted. All patients were older than 18 years and presented with normal hepatic, renal, and bone marrow function. Previous malignancies diagnosed within 5 years (except non-melanoma skin cancer and carcinoma *in situ* of the cervical uterus) were exclusion criteria. All data were recorded according to good clinical practice. The study was approved by the Regional Scientific Ethical Committee (S-20100005) of Southern Denmark and the Danish Data Protection Agency and all methods were performed in accordance with relevant guidelines and regulations. Written informed consent was obtained from all patients enrolled in the study.

### Treatment and evaluation

Treatment consisted of capecitabine 1000 mg/m^2^ twice daily on days 1 through 14 (28 doses) in a 21-day cycle, and oxaliplatin 130 mg/m^2^ as a 2-hour intravenous infusion on day 1. Bevacizumab was given as an infusion (7.5 mg/kg) on day 1 of each treatment cycle. Treatment continued until disease progression or unacceptable toxicity, but treatment-free intervals were accepted after six cycles, if requested by the patient. Response evaluation was based on clinical and radiologic examination using CT scans of the chest and abdomen every 9 weeks and assessed according to the RECIST 1.1 criteria^25^. Responding patients were classified as having complete response (CR) or partial response (PR), while non-responding patients were classified as stable disease (SD) or progressive disease (PD).

### Sampling

Sampling of peripheral blood was carried out at baseline before treatment initiation (serum and whole blood) and then 3-weekly at each clinical evaluation (serum) until progression. To improve comparability, three samples were chosen for the present study (baseline, first clinical evaluation, and progression). The first two were available for analysis from all 88 patients and the sample at progression from 74 patients (84%).

Venous blood, drawn from the antecubital area and collected in 6 ml dry glass, was left for a minimum of 30 min. for a clot to form, spun down for 10 min. at 2500 g, and serum was transferred to Greiner tubes (SIGMA-ALDRICH, USA) and frozen at −80 °C. Blood intended for SNP analyses was collected in 3 ml EDTA tubes and frozen immediately at −80 °C. The median storage time from sampling to analysis was 2.4 years. Samples were transported on ice from storage to analysis.

### Protein analyses

A sandwich enzyme-linked immunosorbent assay (Cloud-Clone Corp., SEL643Hu, Houston, TX, USA) was used to quantify EGFL7 in serum. Analysis was performed according to the manufacturer’s protocol. In brief, 100 µl of standard or sample was added to each well of a 96-well strip plate, pre-coated with an antibody specific to EGFL7, and incubated for 2 hours at 37 °C. Aspiration was followed by addition of detection reagent A, incubation for one hour at 37 °C, aspiration and three times washing. This step was repeated for detection reagent B with 30 minutes of incubation and five washes. Then substrate solution was added, incubated for 15 minutes at 37 °C, followed by addition of stopping solution and read at 450 nm. Concentrations of EGFL7 were assessed through comparisons with the standard curve and multiplied with the initial dilution factor (4 fold). Samples with concentrations above the standard curve were diluted further (and multiplied accordingly).

All samples were assayed in duplicate and the average was used for comparison with clinical data. The total in-house analytical coefficients of variation (CV) at two levels were 19.2% (high) and 23.4% (low). This is considered acceptable in reference to a study population (interpatient) CV of 95%. All protein concentrations are expressed in ng/ml.

### EGFL7 genotyping

We decided to analyse SNPs in the *EGFL7* gene region in order to test for possible functional influence on EGFL7 protein levels and for possible associations with clinical outcome. The selection of SNPs was based on a tagging SNP strategy aiming for a broad coverage of the *EGFL7* gene region, while simultaneously ensuring a sufficient frequency of the rare allele in the selected SNPs. Based on this, five SNPs were chosen for analyses (Supplementary Fig. 1).

Germline DNA was isolated from whole blood by the Maxwell® method according to the user manual (Promega Corporation, WI, USA) (http://www.promega.com/tbs/tm284/tm284.pdf). Genotyping was performed by PCR analysis using the ABI PRISM 7900 HT fast real-time PCR system (Applied Biosystem, Foster City, CA, USA). Commercial assays functionally tested and validated (LifeTechnologies, Carlsbad, CA, USA) were used. Assay numbers are listed in Supplementary Figure 1. The analysis was performed as previously described^26^. Genotyping was not possible in two patients due to an insufficient amount of DNA. All other samples met the quality value threshold of 98%. Blood samples blinded to patient outcome were successfully processed and analysed at the same institution (Vejle Hospital, Denmark).

### Statistics

We report median values with a 95% confidence interval (CI). The Wilcoxon rank sum test was used for comparison of median values. Fisher’s exact test was used for comparison between categorical parameters, while chi-square statistics were used to test for Hardy-Weinberg equilibrium. Survival functions were estimated by log rank tests and illustrated by the Kaplan-Meier method. Simple Cox regression analysis was used to estimate the hazard ratio of individual potential prognostic variables, and variables with p-values below 0.1 were included in multiple Cox regression analyses. Progression free survival was defined as the time from start of treatment until the first documented tumor progression or death from any cause. The PFS data were censored in 15 cases due to liver resection (N = 14) and radio frequency ablation (N = 1) for liver metastases. Data were censored from the day of the intervention, meaning that these patients contributed with “time” until their respective dates of intervention and the interventions did not count as an “event” in the Cox regression analyses. Overall survival was defined as the time from start of treatment until death of any cause. Adjustment for multiple comparisons was not made. All patients were successfully followed until progression. A two-sided 0.05 significance level was used in all statistical tests. Statistical calculations were carried out using the NCSS statistical software (NCSS Statistical Software, Kaysville, UT 84037, USA, version 2007). P values < 0.05 were considered significant, and all tests were two-sided.

## Electronic supplementary material

Dataset 1
