# Supplementary material for: Prognostic importance of circulating epidermal growth factor-like domain 7 in patients with metastatic colorectal cancer treated with chemotherapy and bevacizumab
Source: Sci Rep. 2017 May 24;7:2388. doi: 10.1038/s41598-017-02538-x (PMC5443778; doi:10.1038/s41598-017-02538-x)
Supplement: Supplementary file 1 — Dataset 1 [file 41598_2017_2538_MOESM1_ESM.doc]

**Prognostic importance of circulating epidermal growth factor-like domain 7 in patients with metastatic colorectal cancer treated with chemotherapy and bevacizumab**

Torben Frøstrup Hansen^1^

Rikke Fredslund Andersen^1^

Dorte Aalund Olsen^1^

Flemming Brandt Sørensen^1^

Anders Jakobsen^1^

**^1^** Danish Colorectal Cancer Center South, Vejle Hospital, Institute of Regional Health Research, University of Southern Denmark, Denmark


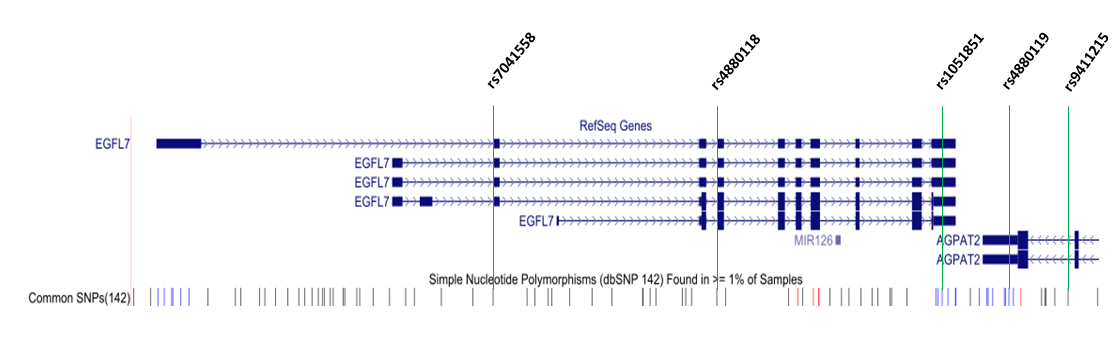


Supplementary Figure S1. The *EGFL7* gene and the first part of the *AGPAT2* gene, illustrating the location of the five analysed SNPs.

Modified from UCSC Genome Bioinformatics, <http://genome.ucsc.edu/>, Kent WJ et al. The human genome browser at UCSC. Genome Res. 2002 Jun;12(6):996-1006.

Assays (LifeTechnologies, Carlsbad, CA, USA)

rs7041558: C___3080937_10

rs4880118: C__25608319_10

rs1051851: C__11571289_20

rs4880119: C___3080939_10

rs9411215: C___1756056_20

Supplementary Table S1. Circulating epidermal growth factor-like domain 7 (cir-EGFL7) at base-line according to patient characteristics.

|  | **Baseline cir-EGFL7**^a^ (ng/ml)  median (95%CI^b^) | **p-value** |
| --- | --- | --- |
|  | N^c^ = 88 |  |
| **Gender** |  |  |
| Male | 334 (165 – 534) | 0.3845 |
| Female | 203 (137 – 396) |  |
| **Age** (years)^*^ |  |  |
| > Mean | 277 (137 – 526) | 1.0000 |
| ≤ Mean | 247 (170 – 400) |  |
| **ECOG** **PS**^d^ |  |  |
| 0 | 195 (142 – 379) | 0.2711 |
| 1-2 | 362 (170 – 534) |  |
| **Tumor resection**^**^ |  |  |
| Yes | 136 (86 – 227) |  |
| No | 504 (203 – 872) | **0.0001** |
| **Localization** |  |  |
| Colon | 362 (170 – 643) | **0.0108** |
| Rectum | 170 (104 – 310) |  |
| Synchronous^***^ | 2301 |  |
| **Metastatic sites** |  |  |
| 1 | 227 (142 – 495) | 0.5287 |
| ≥2 | 271 (170 – 525) |  |
| ***RAS/RAF***^e,****^ |  |  |
| Wild type | 283 (170 – 502)) | 0.555 |
| Mutated | 227 (141 – 400) |  |
| Unknown | 2267 |  |
| **R0**^f^ **resection** |  |  |
| Yes^*****^ | 117 (68 – 227) | **0.0114** |
| No | 336 (195 – 514) |  |
| Cir-EGFL7^a^, circulating epidermal growth factor-like domain 7; CI^b^, Confidence interval; N^c^, Number; ECOG PS^d^: Eastern Cooperative Oncology Group performance status  **^*^**Age at start of treatment  **^**^**Primary tumour previously resected  **^***^**Data from the two patients with synchronous tumours were left out from this analysis.  ^****^Includes KRAS, NRAS, and BRAF mutational status. Data from patients with unknown mutational status were left out from this analysis  **^*****^**Patients resected with a curative intent after initiation of treatment (N=15)  Confidence intervals are not calculated for cases with less than five individuals | | |

Supplementary Table S2. Cir-EGFL7^a^ according to treatment response (N^b^=85*).

|  | **cir-EGFL7** (ng/ml) | | | | | |
| --- | --- | --- | --- | --- | --- | --- |
| **Response** | **Baseline** | **p-value** | **First evaluation** | **p-value** | **Δ**^c^ **baseline / first evaluation** | **p-value** |
| No | 237 (142-525) | 0.836 | 205 (116-288) | 0.527 | 0.59 (0.42-0.75) | 0.755 |
| Yes | 269 (166-466) |  | 153 (105-195) |  | 0.65 (0.44-1.09) |  |
| cir-EGFL7^a^, Circulating epidermal growth factor-like domain 7; N^b^, Number; Δ^c^: change  *Three patients were not evaluable according to RECIST. | | | | | | |

Supplementary Table S3. Genotype distributions.

|  | **Genotype distribution** |
| --- | --- |
| **SNP**^a^ | N^b^=86 (%) |
| **rs**^c^**7041558** |  |
| AA | 13 (15) |
| AG | 41 (48) |
| GG | 32 (37) |
| **rs9411215** |  |
| AA | 7 (8) |
| AG | 34 (40) |
| GG | 45 (52) |
| **rs4880118** |  |
| CC | 72 (84) |
| CT | 14 (16) |
| TT | 0 (0) |
| **rs1051851** |  |
| GG | 57 (66) |
| GA | 28 (33) |
| AA | 1 (1) |
| **rs4880119** |  |
| GG | 3 (3) |
| GA | 24 (28) |
| AA | 59 (69) |
| SNP^a^, Single nucleotide polymorphism; N^b^, Number; rs^c^: reference sequence  The allele frequencies followed the Hardy-Weinberg equilibrium in all cases (p>0.05). Genotyping was not possible for 2 patients due to insufficient amount of DNA. | |

Supplementary Table S4. EGFL7 single nucleotide polymorphisms and the association with cir-EGFL7 protein levels.

|  |  | **cir-EGFL7**^a^ **/** ng/ml | | | | | |
| --- | --- | --- | --- | --- | --- | --- | --- |
| **SNP**^b^ | **Number (%)** | **Baseline** | **p-value** | **First evaluation** | **p-value** | **Progression** | **p-value** |
| **rs**^c^**7041558** |  |  |  |  |  |  |  |
| AA | 13 (15) | 336 (104-949) |  | 118 (59-430) |  | 89 (15-361) |  |
| AG | 41 (47) | 267 (159-502) |  | 164 (105-223) |  | 180 (105-239) |  |
| GG | 32 (37) | 218 (121-400) |  | 181 (72-294) |  | 112 (46-155) |  |
| AA+AG | 54 (62) | 301 (166-502) | 0.419 | 159 (112-229) | 0.827 | 168 (101-226) | 0.052 |
| GG | 33 (37) | 218 (121-400) |  | 181 (72-294) |  | 112 (46-155) |  |
| **rs9411215** |  |  |  |  |  |  |  |
| AA | 7 (8) | 195 (38-949) |  | 85 (23-5000) |  | 44* |  |
| AG | 34 (39) | 247 (142-525) |  | 192 (118-352) |  | 201 (93-246) |  |
| GG | 45 (52) | 271 (137-514) |  | 162 (77-212) |  | 137 (81-182) |  |
| AA+AG | 41 (47) | 227 (146-495) | 0.942 | 168 (112-294) | 0.387 | 125 (72-226) | 0.866 |
| GG | 46 (53) | 271 (137-514) |  | 162 (77-212) |  | 137 (81-182) |  |
| **rs4880118** |  |  |  |  |  |  |  |
| CC | 72 (84) | 197 (146-396) |  | 153 (112-205) |  | 124 (93-173) |  |
| CT | 14 (16) | 335 (195-949) | 0.169 | 260 (83-430) | 0.150 | 201 (50-521) | 0.197 |
| TT | 0 (0) |  |  |  |  |  |  |
| **rs1051851** |  |  |  |  |  |  |  |
| GG | 57 (66) | 227 (165-336) |  | 175 (129-265) |  | 137 (81-194) |  |
| GA | 28 (32) | 348 (142-526) |  | 115 (83-247) |  | 115 (59-198) |  |
| AA | 1 (1) | 991* |  | 168* |  | 697* |  |
| GG | 57 (66) | 227 (165-336) | 0.493 | 175 (129-265) | 0.408 | 137 (81-194) | 0.804 |
| GA+AA | 29 (33) | 362 (142-526) |  | 118 (83-223) |  | 121 (59-198) |  |
| **rs4880119** |  |  |  |  |  |  |  |
| GG | 3 (3) | 195* |  | 116* |  | 83* |  |
| GA | 24 (28) | 349 (97-534) |  | 284 (104-444) |  | 101 (52-246) |  |
| AA | 59 (69) | 237 (165-396) |  | 163 (113-195) |  | 137 (111-193) |  |
| GG+GA | 27 (31) | 336 (104-525) | 0.948 | 247 (104-430) | 0.276 | 101 (52-239) | 0.558 |
| AA | 60 (69) | 237 (165-396) |  | 163 (113-205) |  | 137 (111-193) |  |
| cir-EGFL7^a^, Circulating epidermal growth factor-like domain 7; SNP^b^, Single nucleotide polymorphism; rs^c^, reference sequence  Not all sums of percentages equal 100% due to rounding of data  *Confidence intervals are not calculated for cases with less than five individuals | | | | | | | |
